# The Role of Microglia in Diabetic Retinopathy

**DOI:** 10.1155/2014/705783

**Published:** 2014-08-31

**Authors:** Jeffery G. Grigsby, Sandra M. Cardona, Cindy E. Pouw, Alberto Muniz, Andrew S. Mendiola, Andrew T. C. Tsin, Donald M. Allen, Astrid E. Cardona

**Affiliations:** ^1^Vision Health Specialties, 4109 N. Midland Drive, Midland, TX 79707, USA; ^2^Department of Biology, The University of Texas at San Antonio, San Antonio, TX 78249, USA; ^3^College of Optometry, University of Houston, Houston, TX 77204, USA; ^4^Department of Biology, The University of Texas of the Permian Basin, Odessa, TX 79762, USA

## Abstract

There is growing evidence that chronic inflammation plays a role in both the development and progression of diabetic retinopathy. There is also evidence that molecules produced as a result of hyperglycemia can activate microglia. However the exact contribution of microglia, the resident immune cells of the central nervous system, to retinal tissue damage during diabetes remains unclear. Current data suggest that dysregulated microglial responses are linked to their deleterious effects in several neurological diseases associated with chronic inflammation. As inflammatory cytokines and hyperglycemia disseminate through the diabetic retina, microglia can change to an activated state, increase in number, translocate through the retina, and themselves become the producers of inflammatory and apoptotic molecules or alternatively exert anti-inflammatory effects. In addition, microglial genetic variations may account for some of the individual differences commonly seen in patient's susceptibility to diabetic retinopathy.

## 1. Introduction

It is generally acknowledged that central nervous system (CNS) disorders involve microglial activation and that progression and resolution of many diseases depend in part on the actions of microglia. As resident inflammatory cells of the CNS, microglia potentially modulate inflammatory processes. However, microglial functions do not happen in isolation, but in concert with the activities of neurons, glial, and vascular cells. Diabetic retinopathy (DR) is the leading cause of vision loss in individuals 20–75 years of age. It remains a frightening prospect to patients and physicians as the cause(s) remain unclear. While DR has been described classically as a microvascular disease, recent evidence suggests that changes to retinal microglia are an early feature of retinopathy [1–6]. Clinically, DR is classified as nonproliferative (NPDR) and proliferative diabetic retinopathy (PDR). NPDR exhibits damage to retinal vasculature, leaky blood vessels, and associated mobilization of blood components into the retina. Proliferative DR associates with growth of blood vessels on the surface of the retina. Although diabetic macular edema (DME) can cause vision loss in both NPDR and PDR, the severe angiogenesis in proliferative disease can result in retinal detachment with a potential to cause total blindness. The loss of retinal neurons and the loss of contrast sensitivity in diabetes have only recently been described [1–9]. The study of microglia at different stages of diabetes, and its interaction with peripheral leukocytes and retinal cells, is crucial to provide insights into mechanisms of damage and repair. The use of genetic and induced models of diabetes, in combination with histochemical and imaging approaches, has provided instrumental information in the role of microglia during retinal diseases. Understanding the role of microglia in the diabetic retina is essential in comprehending the inflammatory components during disease progression.

### 1.1. Microglial Phenotypes

Immune surveillance is the most common function associated with microglia during healthy and diseased states. Several studies have elegantly shown that microglia are continuously surveying their microenvironment by extending and retracting their highly motile processes [14, 15]. This property is critical to elicit prompt responses to injury or infection. Hanisch and Kettenmann hypothesize that microdamage in the brain such as microaneurism or the incipient demise of a single neuron can be monitored and repairs can be initiated without triggering a more massive activated state [16–18]. Phagocytosis of cellular debris is an important task of microglia and is tightly linked to their immune surveillant behavior. The activation of microglia in response to neural damage involves proliferative, morphological, immunoreactive, and migratory changes [14, 19, 20]. Studies have suggested that the severity of the microglia response is dependent on the gravity of the neural damage; additionally, the microglia response can be either neural-protective or toxic [16, 21–28]. Graded changes in the morphological appearance of microglia are often used to distinguish surveillant or highly ramified microglia from activated or amoeboid microglia. The amoeboid state refers to cells with larger cell bodies and thicker processes and is usually correlated with activation of microglia in response to CNS insults such as autoimmune inflammation, neuronal injury, cancer, infection, or hyperglycemia. In the retina, under normal conditions, surveillant microglia are localized to the inner and outer plexiform layers and are absent from the outer nuclear layer (Table 1) [10, 29]. Retinal microglia activation correlates with neuronal damage induced by retinopathies such as autoimmune reactions, ocular infections, ischemia, neural injury,and cytokine exposure but direct evidence of microglial mediated neurotoxicity is missing [30]. Interestingly, during normal CNS development, microglia possess an amoeboid morphology and as microglia become mature they transition to a ramified form [31]. However, there are instances in which microglial activation occurs without evident cellular transformation. For example, low doses of systemic lipopolysaccharide (LPS) induce the production of proinflammatory cytokines without any apparent change in microglial morphology [32]. Therefore, microglial responses are shaped by an arsenal of signal transduction pathways that lead to an effector phenotype associated with neuroprotection or neurotoxicity. Disregulated microglial responses could be the result of a triggering receptor or the loss of a constitutive inhibitory signal [33, 34]. Ultimately, the balance between these contrasting outcomes guides tissue repair or exacerbates injury.

### 1.2. Hyperglycemia, Inflammation, and Microglia

It has been suspected that chronic inflammation played a role in DR since 1964 when Powell found that high dose aspirin used to treat rheumatoid arthritis was also helpful in treating DR [35]. Several molecular changes associated with chronic inflammation have been detected in the diabetic retina and there is growing evidence that this plays a role in both the development and progression of DR [36–38]. Various mechanisms including ischemia, hypoxia, hyperglycemia, dyslipidemia, advanced glycation end products (AGE), and endoplasmic reticular (ER) stress have been hypothesized to contribute to the inflammatory component of DR [39, 40]. Chronic inflammation has also been linked to diabetic nephropathy and peripheral neuropathy [37]. Since there is significant evidence that chronic inflammation plays a role in diabetic retinopathy and microglia are the resident immune cells in the retina, it is likely that unleashed microglial activation may play a role in the neurotoxicity and tissue damage in the diabetic retina. Microglia may function in acute inflammation protecting the CNS, but have also been linked to deleterious effects on the CNS when they exhibit chronic inflammation in diseases such as HIV, multiple sclerosis, and Alzheimer's [41]. Yang et al. defines DR as a “chronic, low-grade inflammatory disease of the retina” [42]. Since inflammatory conditions present in diabetes can activate microglia, they further state that “activated microglia thereby stimulate a cycle of inflammation that recruits leukocytes, causes vascular breakdown, and directly induces glial dysfunction and neuronal cell death through the release of cytotoxic substances” [42].

It is well established that hyperglycemia results in increased cellular oxidative stress. This can be either through (1) direct generation of reactive oxygen species (ROS), (2) alteration of the oxidation-reduction balance, that is, increase of polyol pathway flux reducing NADPH reservoirs and/or conversion of oxidized glutathione to glutathione, (3) increase of AGE formation, protein kinase C (PKC), or (4) superoxide overproduction by the mitochondrial electron transport chain [43–45]. It is also known that hyperglycemia induced ROS can cause nuclear factor kappa B (NF-*κ*B) translocation to the nucleus [46, 47]. Many of these pathways work to elevate NF-*κ*B, which when activated produces many inflammatory cytokines including tumor necrosis factor alpha (TNF-*α*), interleukins 1 beta, 6, and 8 (IL-1*β*, IL-6, IL-8), vascular cell adhesion molecule 1 (VCAM-1), and intercellular adhesion molecule 1 (ICAM-1) [43, 48–50]. Wautier et al. found increased AGE formation on the erythrocytes of human subjects with diabetes, which, when cultured with human umbilical cord endothelial cells, caused an increase in the generation of ROS and activation of NF-*κ*B. This increase in ROS was reversed by antioxidants [46, 51, 52]. Such soluble mediators have the potential to activate perivascular microglia [50]. Furthermore, it is known that AGE concentration increases in the retina during diabetes [50, 53]. Wang et al. found that AGE exposure activated microglia, as measured by NF-*κ*B translocation, increased ROS production and promoted TNF-*α* mRNA production and release of TNF-*α* from retinal microglia [50]. The increase in ROS can also locally damage vessels. TNF-*α* was found to enhance the expression levels of adhesion molecules such as ICAM-1 and VCAM [54]. Therefore, TNF-*α* production may trigger leukocyte infiltration to retinal vessels with resulting vascular inflammation [50]. The breakdown of the blood-retinal barrier (BRB) is an early development in the progression of DR [55]. This leads to increases in AGE concentration throughout the retina, resulting in vessel and neuronal damage [50, 55]. Increased exposure of inflammatory agents, such as AGE to the microglia, as a result of early BRB breakdown, results in activation of perivascular microglia. This activation leads to a release of microglial products further breaking down the BRB and an increase in retinal chronic inflammation [50, 56, 57]. However, the contribution of hematogenous cells such as neutrophils, T cells, or B cells is still yet to be determined.

As previously mentioned nonenzymatic glycoxidation of proteins, AGE formation, is elevated in oxidative stress including diabetes [46, 58]. AGE formation involves the irreversible glycation of long-lived tissue proteins which can alter their conformations and their physiological functions [59]. The receptor for AGE (RAGE) is a member of the cell surface molecule immunoglobulin superfamily which responds to multiple ligands including AGE, amyloid, amphoterin, and S100/calgranulins. The receptor tends to accumulate where the concentration of its ligand is the highest and contains a short cytosolic tail, which appears to be crucial for cellular activation, and therefore has important functions in signal transduction. Leakiness in bovine aortic endothelial monolayers induced by diabetic red cells and blood brain barrier vascular permeability in streptozotocin-induced (STZ) diabetic rats was reduced by inhibiting the AGE-RAGE interaction using either anti-RAGE antibody or soluble RAGE (sRAGE) which lacks the transmembrane portion [46, 52]. This permeability was also blocked with the antioxidants vitamin E and probucol [52]. Wautier et al. report that AGE-RAGE binding generates ROS through activation of NADPH oxidase [60]. Furthermore, RAGE expression has been found in microglia [61, 62]. Wong et al. report that AGE-RAGE induces microglial activation and oxygen free radicals serve as second messengers in AGE-RAGE inflammatory signal transduction pathways [63]. AGE-RAGE interactions have been linked to tumor angiogenesis. Thus, it is reasonable that RAGE-mediated cellular interactions of chronic inflammation and angiogenesis render tissues susceptible to diabetic complications [46].

## 2. Microglia in Human and Animal Models of Diabetic Retinopathy

### 2.1. Histopathological Evidence of Microglial Involvement in Diabetic Retinopathy

Microglia activation in different forms of DR has been studied in healthy controls and DR [64]. Microglia labeling with antibodies against human microglial markers CD45, CD68, and HLA-DR, a major histocompatibility complex II antigen, was evaluated in individuals with no diabetes (Table 2), background (Table 3), pre-proliferative (Table 4), proliferative DR (Table 5), and diabetic macular edema (DME) [64]. Microglia were generally increased in number (proliferation) and in size and found mainly associated with retinal vasculature, dilated veins, cotton-wool spots, and around microaneurisms in the inner retina. The eyes with proliferative retinopathy had an increase in activated microglia in the ganglion cell layer (GCL) and the neovascular area of the vitreous. In the DME eyes, proliferated microglia were found throughout the retina and subretinal space [64].

### 2.2. Morphological and Molecular Studies of Retinal Microglia in Various Animal Models of DR

Zeng et al. using STZ-induced diabetic rat model propose an association between microglia and neuronal cells [6]. After 1 month of diabetes symptoms, microglia appeared hypertrophic and their numbers had increased significantly. Interestingly the number of microglia peaked at 4 to 6 months in the inner plexiform layer (IPL) coinciding with the decrease in the number of neuronal cells. Additionally, microglia were observed in the outer plexiform layer (OPL) at 4 months, but at 14–16 months, activated microglia were observed also in the outer nuclear layer (ONL) and photoreceptor layer (PRL) of the outer retina. The authors suggest that these microglia were attracted to these areas by damage to neuronal cells and photoreceptors, respectively. Initial signals eliciting microglial activation within the inner retina were likely from cell death in the nerve fiber layer (NFL) and inner nuclear layer (INL) [6]. Also the activation, proliferation, and trafficking of microglia in both the inner and outer retina in the Goto-Kakizaki rat model (GK) has been reported [65]. Interestingly, Omri et al. found the development of transcellular pores lined with occludin, caveolin-1 (CAV-1), and protein kinase C zeta (PKC *ζ*) in RPE cells which reached a maximum at 6 months of hyperglycemia in streptozotocin (STZ) induced diabetic mice. By 12 months the number of pores is reduced in diabetic mice, but increased non-diabetic controls. Interestingly, this reduction correlated with an increased population of microglia/macrophages in the outer retina and also with microglial migration through a disorganization of the intracellular RPE tight-junctions. Rhodamine-liposomes were injected in the vitreous helping to identify microglial migration through the retina into the choroid. A PKC inhibitor reduced accumulation of microglia and macrophages implicating PKC *ζ* as a factor in the diabetic retina and suggests that manipulation of microglia activity might be therapeutic in the treatment of diabetic retinopathy. Furthermore, while retinal microglia express MHC class II markers and ramified microglia have many features similar to ocular dendritic antigen-presenting cells (APCs), there remains doubt as to whether they can function as antigen presenting cells [10, 66, 67].

The cooperative function of microglia and hematogenous macrophages was defined more recently in the removal of dead photoreceptors resulting from light damage in susceptible (albino) mice models. Joly et al. exposed mice injected with green fluorescent protein (GFP) positive bone marrow cells or GFP-labeled microglia to focal blue light [68]. Blood borne macrophages entered the retina via the optic nerve and ciliary body and migrated to the damaged area. Resident microglia were activated throughout the retina but assumed the phagocytic phenotype only at the site of photic injury. The macrophages, which had ingested debris, including rhodopsin from photoreceptor outer segments, were observed to leave the retina via the optic nerve head. The authors suggest that the departing macrophages could reach the spleen and raise an immune response against specific retinal proteins.

The role of microglia in initiating chronic neuroinflammation in the diabetic retina has been recently reviewed by Abcouwer [69]. Once activated, microglia can assume any of the several phenotypic states, some inflammatory, others anti-inflammatory. In diabetic retinas, macrophages of hematogenous origin are also present in damaged portions of the retina. Future research should aim to distinguish the relative importance of these two retinal monocytic populations in the chronic inflammatory response to diabetes. Therapeutic approaches to diabetic inflammation and homeostasis could employ signals to modify microglial and/or macrophage function.

## 3. Molecular Mechanisms Linking Hyperglycemia and Microglial Activation

### 3.1. Classic Molecular Mechanisms

Cytokines considered markers of inflammation in DR include TNF-*α*, IL-1, IL-6, IL-8, CRP, and chemokine (C-C motif) ligand 2 (CCL2). These cytokines are produced chiefly by activated immune cells, although resident glial cells such as astrocytes have the potential to also secrete a wide array of chemokines [37, 70]. Cyclooxygenase (COX) enzymes whose end-products are prostaglandins, thromboxanes, and leukotrienes have also been associated with DR [37, 71, 72]. In fact, thromboxanes and prostaglandins have been associated with the development of angiogenesis [37, 71, 73].

It is well known that elevated levels of IL-1, IL-6, gamma-interferon (*γ*-IFN), and TNF-*α* cytokines activate microglia [56, 74]. Zorena et al. report that higher than normal serum levels of CRP, IL-6, and TNF-*α* have been found in children with type 1 diabetes and NPDR [37, 75]. Several studies have given conflicting reports over the role of CRP in DR [76–82]. Correlations to plasma levels of TNF-*α*, IL-1*β*, IL-8, and CCL2 and vitreous levels of TNF-*α*, IL-1, IL-6, IL-8, and CCL2 have been found for both NPDR and PDR [37, 38, 83–86]. In children and adolescents with type 1 diabetes, serum TNF-*α*, vascular endothelial growth factor (VEGF), AGE concentration, and urinary albumin excretion were all predictive of retinal microvascular disease [87]. Similarly Ben-Mahmud et al. found a correlation between the levels of plasma TNF-*α* and the degree of DR in patients with either type 1 and type 2 diabetes [84]. Diabetic retinopathy is typically treated as a vascular disease, and thus proangiogenic factors such as vascular endothelial growth factor (VEGF) have been shown to be elevated in DR rodent models and in the vitreous of human patients leading to increased leakage through retinal vascular walls [88, 89].

Molecules released by activated retinal microglia include glutamate, proteases, leukotrienes, IL-1*β*, IL-3, IL-6, TNF-*α*, VEGF, lymphotoxin, macrophage inflammatory protein 1 (MIP-1), matrix metalloproteinases (MMPs), and other ROS (Table 6) [56, 57, 92–97]. Glutamate, proteases, leukotrienes, IL-1*β*, IL-6, TNF-*α*, VEGF, lymphotoxin MMPs, and ROS have all been linked to diabetic retinopathy and a role for MIP-1, IL-1, and IL-3 in angiogenesis has been established in ischemic mouse models [56, 85, 98–102]. IL-1*β* is a trigger of the neuroinflammatory cascade [90, 103]. Wang et al. found increased production of IL-1*β*, TNF-*α*, and nitric oxide (NO) produced into the culture medium by rat retinal microglia that had been stimulated with LPS [57]. Intracellular calcium levels can regulate release of microglia inflammatory mediators such as IL-1*β*, TNF-*α*, and NO [104]. De-Oliveria-Simoes-Pereria et al. found that when rat retinal cells were cultured in high glucose conditions, purinergic P2 receptors in microglia were upregulated, causing a calcium influx and release of additional microglial and associated proinflammatory mediators seen in the early stages of DR [104]. This release of inflammatory mediators and neurotransmitters may also contribute to early retinal neuron death associated with diabetes [104]. TNF-*α* and IL-1*β* are known to increase caspase 3 activity, inducing endothelial cell apoptosis [40, 105, 106]. Microglia are considered the early primary source of IL-1 in CNS injury, infection, or inflammation. Cardona et al. found that systemic inflammation induces IL-1 as a mediator of microglial neurotoxicity in mice lacking the inhibitory fractalkine receptor [103, 107]. While Liu et al. found lack of IL-1*β* production from brain microglial cultures in hyperglycemia, Yang et al. found increased retinal production of IL-18, TNF-*α*, and IL-1*β* in hyperglycemic rats which was reduced with the anti-inflammatory and suppressor of microglia baicalein [42, 90].

Yoshida et al. found activated NF-*κ*B in pericytes, vascular endothelial cells, macrophages, and microglia in a C57BL/6N hypoxia-induced mice model of neovascularization [49]. They also propose that NF-*κ*b activation is required for retinal angiogenesis and that inhibition of NF-*κ*b could be used to ameliorate neuronal cell death in PDR [49]. Inhibition of NF-*κ*b by the antioxidant resveratrol, a naturally occurring phenol that increases the action of superoxide dismutase (SOD), resulted in decreased serum levels of IL-1*β*, IL-6, and TNF-*α*. However, the direct correlation between NF-*κ*b inhibition and neuronal damage has not been addressed [43, 48].

Microglia in rat retinas become activated soon after the onset of hyperglycemia and some studies indicate that inhibition of microglial activation correlates with neuronal protection in the diabetic retina [2, 6, 56]. Krady et al. found the microglia at this early stage in the inner retina become transformed to an activated amoeboid state, with few or no processes, corresponding to an increase in retinal expression of IL-1*β* and TNF-*α* mRNA [56]. They found a 6-fold increase in TNF-*α* mRNA and a 4.5 fold increase in IL-1*β* mRNA. Diabetic rats treated soon after STZ injection with minocycline did not show this increase in IL-1*β* and TNF-*α* mRNA. In addition, in microglial cell culture the addition of minocycline prevented an increase in the expression of COX-2 mRNA induction by TNF-*α* and caspase 3 activation was blocked with a resulting decrease in retinal neuron death [56]. It is known that minocycline has anti-inflammatory effects separate from its antimicrobial effects [108]. Minocycline is a semisynthetic tetracycline derivative found to cross the blood-brain/retinal barrier and inhibit microglial proliferation and activation by inhibiting the p38 MAPK pathway [109]. Minocycline inhibits matrix MMPs, nitric oxide synthases (NOS), COX-2, phospholipase A2, and (IL-1*β*-) converting enzyme (ICE) mRNA, each of which is thought to be produced by activated microglia [109].

Microglia are known to produce COX-2 [56, 110]. As mentioned earlier, the use of COX inhibitors such as aspirin in diabetes has had questionable results with the early work of Powell showing promising results [35, 111]. The Dipyridamole Aspirin Microangiography of Diabetes Study Group (DAMAD) in 1989 found a small reduction in the number of microaneursyms (MAs) in an aspirin treated group (990 mg daily), while the Early Treatment of Diabetic Retinopathy Research Group (ETDRS), in 1991, found no significant difference (650 mg daily) [112]. ETDRS found no significant help from aspirin usage in the development of high-risk DR, the risk of vision loss, or the development of vitreous hemorrhage [113]. Kern and Engerman found a significant inhibition in the development of acellular capillaries and retinal hemorrhages in diabetic dogs using 40 mg/kg/day aspirin (considered the maximum dose for dogs), but insignificant effect on the number of MAs or pericyte ghosts [111]. Acellular capillaries lose their pericytes and then their endothelial cells, leaving empty tubes formed by basement membrane before eventually losing perfusion [114]. Talahalli et al. found increased leukotriene production from retinal “glial” cells only when stimulated by precursor exogenous leukotriene from bone marrow derived cells. Whole retina lysates from diabetic mice exhibited significantly more LTA_4_ hydrolase, which converts leukotriene A (LTA_4_) to leukotriene B (LTB_4_), than nondiabetic mice [115]. Therefore the use of nonsteroidal anti-inflammatory drugs (NSAIDS) is questionable for long term management of DR.

The hypothesis that glycated products trigger cytokine release by microglia and initiate a circle of inflammation is supported by the following observation. Using STZ induced diabetic rats, TNF-*α* colocalized to Iba-1+ microglia but not to Muller cells or astrocytes [116].* In vitro* cultures showed that TNF-*α* production was induced by glycated albumin and cytokine production and blocked by the ERK and p38MAPK inhibitors [116]. The link between increased TNF-*α* serum levels and activation of the glycosylating enzyme 2GlcNac-T provides evidence of the complex pathways induced by hyperglycemia [99]. The involvement of glycation and enzyme-mediated glycosylation therefore awaits further investigation.

The role of NO in the diabetic retina is still not clear. NO can be generated from L-arginine by catalysis of reactions involving nitric oxide synthase expressed by neurons (nNOS), endothelial (eNOS), and induced on inflammatory cells (iNOS). NO regulates vascular tone and can also react with superoxide to form peroxynitrate which can damage proteins, lipids, and DNA. Increased expression of iNOS has been found in diabetic retinas of human patients and in rodent models [117]. Blocking iNOS with aminoguanidine inhibited AGE formation and decreased hyperglycemia induced microvascular anomalies in dogs, rats, and mice [35, 36, 59, 118, 119]. Diabetic iNOS-deficient mice exhibited decreased capillary degeneration, pericyte ghost, superoxide production, and reduced leukostasis [117]. Increased eNOS expression has also been associated with retinal vascular complication in diabetic mouse models [120]. Therefore, the effects of iNOS and eNOS in the retina may act synergistically to promote chemotaxis and tissue damage and may explain the pathology associated with extravasation in PDR.

### 3.2. Other Modifiers of Retinal Inflammation

Pigment epithelial-derived factor (PEDF), a protein exhibiting both antiangiogenic and neuroprotective properties, has been shown to effectively modulate diabetic retinal complications such as neurodegeneration, inflammation, and microvascular insult. Furthermore, when topically applied to Ins2^Akita^ mice, the PEDF peptide PEDF78-121 (P78) prevented microglia activation and retinal ganglion cell dropout, ultimately leading to vascular stability. Insulin-2 Akita (Ins2^Akita^) have been used as a model of type 1 diabetes due to a mutation in the insulin 2 gene that leads to an abnormal protein structure and aggregation leading to death of pancreatic beta cells [121]. It is well established that the Ins2^Akita^ mouse model develops significant retinal pathology as shown by quantifiable vision deficits in an optomotor behavior [122]. Several cytokines, IFN-*γ*, TNF-*α*, IL-3, IL-6, IL-10, and IL-12, were increased in the vitreous of diabetic mice (see Table 6). P78 reduced the quantity of all of these cytokines, while another PEDF peptide, PEDF60-77 (P60), reduced all but IL-2 and TNF-*α* [91].

Among other mediators the chemokine fractalkine (CX3CL1 or FKN) has been shown to modulate microglial function. FKN exists as a cell membrane-bound or soluble protein [123]. In the CNS it is constitutively produced by neurons while its receptor CX3CR1 is only expressed by microglia [107, 124]. FKN and its receptor play an integral role in the process of both inflammation and neurotoxic responses [107]. Cardona et al. report that CX3CR1* in vivo *“governs critical components of the microglial response to systemic inflammation” [107]. Hatori et al. report in cell culture the application of nanomolar concentrations of soluble CX3CL1 to the treating media increases the proliferation of microglia 3 fold [124]. In addition, a deficiency of microglial CX3CR1 induces neurotoxicity and an increase in inflammatory cytokines such as IL-1*β*, IFN-*γ*, and IL-17 [107, 125]. Diabetic mice with microglial CX3CR1 deficiency exhibit increased numbers of vitreal and subretinal macrophages and also showed severe morphological retinal microglial network abnormalities [126]. While the precise role of CX3CR1 on the retina remains unclear, four allelic variants of this gene have been found in 20–30% of the population. One of these variants, the M280, has been associated with enhanced susceptibility to age-related macular degeneration (AMD) [127–129]. Does this variant also result in higher susceptibility to DR?

### 3.3. Genetic Variations in Microglial Responses

Gene expression profiling has offered clues as to the potential regulators of the effector responses of microglia. Some of the most interesting potential targets are chemokines and other myeloid specific transmembrane receptors. The team of immunologist and computational biologist of the Immunological Genome Project (ImmGen) performed gene expression and regulatory networks analyses in tissue resident macrophages [130]. They reported a high degree of diversity in gene expression among dendritic cells and macrophages isolated from peritoneum, spleen, lung, and microglia. A few of the transcripts that were upregulated in microglia and linked to diabetes are discussed.* Serpinf1* (serpin peptidase inhibitor, clade F) or PEDF is reported to promote neuronal survival and differentiation and to be a potent inhibitor of angiogenesis [131]. Plasma levels of * secreted protein acidic and rich in cysteine (SPARC)* associated with insulin resistance, dyslipidemia, and inflammation during gestational diabetes mellitus [132]. Since SPARC is a widely expressed profibrotic protein that modulates tissue physiology by altering cell-extracellular matrix interactions, it has the potential to mediate microglia cell proliferation and migration.* IL-7r* was studied in a Spanish cohort of patients with type 1 diabetes and the homozygous IL-7r allele rs1445898 showed a trend towards a protective effect based on the genotype and an association to early onset diabetes [133]. The* adenosine A3 receptor* (Adora3) binds adenosine, a phosphohydrolytic derivate of ATP, and mediates microglial migration [134, 135]. Also, A2A adenosine receptor engagement led to TNF-*α* release by retinal microglia, and treatment with A2AAR antagonist resulted in a marked decrease in diabetes-induced retinal cell death [136, 137].

In conclusion, the ImmGen study provides novel information to further our understanding of microglia regulation during retinopathies. Several genes have been associated with susceptibility to eye diseases but how genetic backgrounds affecting microglia effect functions, neuronal damage, and inflammation awaits further investigation.

## 4. The Effects of Diabetes Treatments on Microglia

The nature of retinal microglia under normal and reactive conditions, and how phenotypic transformations affect microglia function, is not well understood. Recent studies have focused on the effects that current therapies such as laser photocoagulation and growth factor inhibitor injections have on microglia. In this section we discuss recent findings regarding the effects of light induced retinal treatments and the use of anti-VEGF therapy, steroids, and tetracycline inhibitors.

### 4.1. Light-Induced Retinal Damage and Laser Photocoagulation

It is known that prolonged exposure to bright light induces retinal damage and photoreceptor degeneration [138–140]. However, the effects that light induced photoreceptor degeneration has on microglia cells are not well understood. A study conducted by Santos et al. demonstrated that microglia were activated in the retina of mice with light induced photoreceptor degeneration. Microglia were shown to change from a ramified to an amoeboid morphology and to migrate from inner retinal layers to the outer nuclear layer. Additionally, the activated retinal microglia expressed CD11b, CD45, F4/80, and SRA consistent with immunophenotypic activation [141]. Additionally, increases in signal peptides such as CCL2, MIP-1*α*, and TNF-*α* have been noted in a study of light-induced photodegeneration. These signals or their analogs induce apoptosis of photoreceptor cells in the ONL [26, 142]. The dynamic activation of microglia in response to light induced retinal degeneration suggests a damaging inflammatory response at the photoreceptor cell layer. However, microglia can also be neuroprotective thus the nature and function of these cells after light induced retinal damage remain unclear.

Laser retinal photocoagulation is routinely used in the clinical setting for treatment of eye diseases such as proliferative diabetic retinopathy. Laser retinal treatment delivers varied degrees of coagulation through the retinal thickness; these vary from mild, moderate, or heavy depending on the retinal layers involved. The objective is to use light energy to cauterize blood vessels, coagulate tissue to reduce oxygen consumption, and prevent the formation of new vasculature and its effects, such as retinal detachment. However, the effect of retinal photocoagulation on microglia activation has not been determined. A study using* ex vivo* live retinal imaging in conjunction with laser treatments describes microglial behavior in response to focal retinal injury. In this study, 50–100 *μ*m photocoagulation injuries were induced using an argon laser platform in mice retina explants. Using time-lapse confocal microscopy, microglia were observed to change from a ramified, symmetrical, and arbor-shaped cell to a polarized morphology. The microglia morphology was observed to become less branched and extended fewer but longer processes toward the site of injury; these microglia were also reported to acquire migratory drive toward the injury. Additionally, other activated microglia were reported to gain an amoeboid morphology and migrate toward the laser induced retinal injury, and some microglia were observed to maintain their normal ramified arbor morphology [143]. The observations in this study suggest that retinal microglia may be in multiple states of activity and may transform from being at rest to a dynamic active state after focal laser treatment.

In a report by Song et al., the activation of microglia and the intracellular pathways involved in activation were studied in response to low-level laser therapy (He-Ne 632.8 nm, 64 mw/cm^2^). Using a tissue culture system, the investigators showed that low-level laser therapy could activate Src and may lead to microglial phagocytic activity. Additionally, it is suggested that activation of Src kinases may downregulate the expression of proinflammatory cytokines and NO. In this experiment, low-level laser therapy was delivered to cultured SH-SY5Y neuroblastoma cells. Thus it is possible, as the author mentions, that the microglia in the culture system may have responded to malignancy markers, in addition to the laser treatment [144]. Thus, to determine the effects of low-level laser therapy in a more physiological system, future studies could benefit from the use of primary neuronal or microglial cultures and* in vivo* photocoagulation models of variable degrees.

### 4.2. Anti-VEGF Injections

It is well documented that blocking VEGF has inhibitory effects on neovascularization. However, the effects that blocking VEGF receptors have on retinal microglia are not well understood; recently, studies have begun to elucidate information regarding the control of microglia infiltration by targeting VEGF receptors. Huang et al. demonstrated that blocking of the chemoattractant receptors VEGFR1 and VEGFR2 reduced microglia/macrophage infiltration in laser-induced choroidal neovascularization (CNV) [145]. This report shows that VEGFR1 and its endogenous ligands were expressed early in the CNV development process, while VEGFR2 expression appeared in late stages. Additionally, blocking of VEGFR1 inhibited infiltration of activated microglia at early and late stages of CNV, while VEGFR2 was also reported to inhibit microglia infiltration but only at the late stage of CNV development [145]. This study suggests a potential use for VEGF receptor blockers and their inhibition of the microglia inflammatory response as a therapeutic approach.

### 4.3. Steroid Therapy

Corticosteroids have potent anti-inflammatory effects and have a long history of use in the eye. Given the apparent role of inflammation in the pathogenesis of DR and DME, intravitreal steroids have been utilized for the treatment of DME. The mode of action is unclear, but it has been suggested to be through multiple mechanisms including their ability to inhibit expression of VEGF [146, 147]. The effects of steroids on microglia under DR and DME conditions remain unknown; however, other ocular disease models are starting to elucidate the effects of intravitreal corticosteroid delivery on microglia.

For example, in a study focused on N-methyl D-aspartate (NMDA) induced glaucoma, Singhal et al. observed a reduction in the number of microglia induced by the loss of the retina ganglion cells when triamcinolone acetonide (TA) was used along with the NMDA. Then Muller cells from the Muller cell line MIO-M1 were transplanted into the eyes of the glaucoma model. A reduction of transplant induced activated microglia was observed in eyes that had been treated with TA [148]. In a separate study, Shen et al. delivered TA by intraocular injection to a transgenic model which mimics the type of damage that might occur in AMD or DR. In this model the results of Muller cell ablation are retina degeneration with photoreceptor loss, vascular leaks, and retinal neovascularization. This study reported that TA prevented photoreceptor degeneration and inhibited activation of microglial and Muller cells. Additionally, TA attenuated Muller cell loss and inhibited overexpression of p75 neurotrophin receptor (NTR), TNF-*α*, and proneurotrophin (NT) and the activation of p53 and p38/stress-activated protein kinase (SAPK) signaling pathways. It is unclear whether TA inhibited these reactions from microglia, Muller cells, or both [149]. Treatment with TA also prevented the development of retinal vascular lesions and inhibited fluorescein leakage from established vascular lesions. Furthermore, in a study using retina degeneration model, S334ter-line-3, the corticosteroid fluocinolone acetonide (FA) was implanted and delivered to the vitreous. Electrophysiology results from this study show that the ERG a and ERG b waves are preserved in experimental eyes over nontreated controls. The thickness of the ONL was also reported to be between 22% and 25% higher in the eyes that were treated with the corticosteroid. Finally, changes in microglia cells were examined on retinal whole mounts. Activated microglia were found in the ILM and the photoreceptor cell layer. The activated microglia count was reported to be 4 times lower in the FA treated eyes than the control samples [150]. Thus the effects of corticosteroids on microglia seem to be of those of an anti-inflammatory agent preventing microglia activation, attenuation of proinflammatory mediator expression, and modulation of immune signals. Cortisol is known to reduce the activated microglial expression of iNOS mRNA as well as reducing the amount of iNOS, NO, COX-2, and TNF-*α* [150] Additionally, corticosteroids have a protective effect, which may be the result of their ability to attenuate overexpression of VEGF and transcription factors that regulate proapototic genes.

### 4.4. Inhibition of Microglia as a Therapeutic Therapy

As mentioned previously, the tetracycline derivatives minocycline and doxycycline have been linked to inhibition of inflammation and prevention of microglial activation in the retina [56, 151, 152]. Recent clinical trials have investigated the inhibition of microglia as a method to inhibit DR. Cukras et al. performed a prospective, nonrandomized, and uncontrolled single center pilot study to evaluate the use of oral minocycline on 5 human subjects with DME that had previously had focal laser photocoagulation, but in whom DME had persisted [153]. At six months they found a modest increase in best-corrected visual acuity (BCVA), a 5–10% reduction in central subfield retinal thickness (CST) on optical coherence tomography (OCT), and a decrease in the area of late leakage on fluorescein angiography (FA) [153]. The authors note that this improvement, though small, compared favorably with the control arm of the Ranibizumab in Diabetic Macular Edema (RESOLVE) study at 6 months [153, 154]. Recently, Scott et al. performed a randomized, double-masked, 24-month clinical trial using doxycycline monohydrate orally on 30 patients who had at least one eye with severe NPDR or PDR eyes rated as ETDRS less than high risk [113, 155]. While they found no significant difference between those taking the doxycycline and the control group on white/white visual field testing, contrast sensitivity, visual acuity, or anatomic factors, foveal frequency doubling perimetry (FDT) was improved at 6, 12, and 24 months after treatment [155]. Foveal FDT has been determined to be an accurate measure of inner retinal function and has high diagnostic test sensitivity for subjects with NPDR and PDR [156]. However, another randomized, double-masked, 24-month clinical trial using doxycycline monohydrate in 33 patients with mild to moderate NPDR reported no significant difference between control and treatment groups using the previously mentioned test including foveal FDT [113, 157]. Studies with an increased number of patients may clarify in the future the broad application of these treatments. Therefore, there is an urgent need to find additional therapeutic alternatives for the management of DR.

## 5. Conclusions and Perspectives

**Figure 1:**
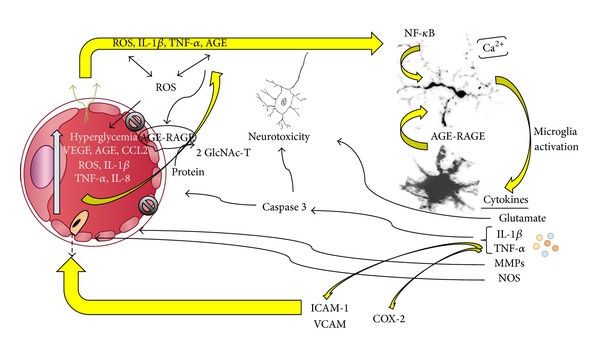
Inflammation during diabetic retinopathy. Increased plasma levels of blood glucose, vascular endothelial growth factor (VEGF), advanced glycation end-products (AGE), reactive oxygen species (ROS), chemokine (C-C motif) ligand 2 (CCL2), interleukins 1 beta and 8 (IL-1*β* and IL-8), and tumor necrosis factor alpha (TNF-*α*) profuse through leaky capillary endothelial cell junctions by the actions of VEGF. IL-1*β*, AGE, ROS, and TNF-*α* activate microglia to produce glutamate, matrix metalloproteinases (MMPs), nitric oxide synthases (NOS), IL-1*β*, and TNF-*α*. IL-1*β* and TNF-*α* drive the production of caspase 3, which along with glutamate is neurotoxic to retinal ganglion cells. Caspases also damage capillary endothelial cells and pericytes. TNF-*α* leads to production of ICAM-1 and VCAM that help recruit macrophages through the capillary walls sustaining a chronic inflammatory response. COX-2 is also a product stimulated by IL-1*β* and TNF-*α*.

Many have proposed that chronic inflammation, including cytokine and chemokine release, cell death, increased vascular permeability, neovascularization, and repair attempts play a role in DR, not only in early changes, but also during the late proliferative stage. It is now established that the loss of retinal ganglion cells occurs early in the DR process. Microglia have been linked to neuronal loss in several neurodegenerative diseases by a mechanism of chronic inflammation. Therefore, it is probable that disregulated microglial activation also functions in the loss of these retinal cells. We propose that some of the first signals of hyperglycemia are picked up by the perivascular microglia from capillary blood or the inner retinal parenchymal microglia, which are exposed from the vitreous. This may be either from hyperglycemia itself or from a systemic deluge of inflammatory cytokines, AGE, or ROS which have been found to increase in the capillary blood and vitreous (Figure 1). The effects of VEGF may elevate exposure to inflammatory mediators in the retinal capillaries. This leakage leads to microglial activation resulting in production of glutamate, IL-1*β*, TNF-*α*, MMPs, and NOS. IL-1*β* and TNF-*α* lead to production of caspase 3, which along with glutamate, results in neurotoxicity of retinal ganglion cells. Caspase 3 also can be toxic to retinal capillary endothelial cells and pericytes. TNF-*α* leads to production of ICAM-1 and VCAM, leading to increased leakage of macrophages through capillary walls, sustaining chronic inflammation. COX-2 is also a product of IL-1*β* and TNF-*α* stimulation. MMP remodeling of capillary basement membranes can further play a role in vessel destruction. Activation of NF-*κ*B as well as its release of inflammatory cytokines is linked to both early and late stages of diabetic retinopathy. Stages of the inflammatory pathway mediated through ROS, AGE-RAGE, NF-*κ*B blockers, or genetic manipulation of fractalkine could be targeted to reduce chronic activation and retinal translocation of microglia in the diabetic retina.

It has also become obvious that people react differently to hyperglycemia. Some develop extremely morbid cases of DR and some seem to go unscathed. It is well established that control of systemic factors such as blood glucose level and blood pressure plays significant roles, but genetic factors likely alter the variability of DR morbidity when systemic factors seem to be similar. As pointed out in this paper, certain genetic variations may regulate the microglial responses to diabetes. Certainly, other yet unknown genetic factors are likely playing a role in this degenerative process.

Microglial responses to treatment of DR including anti-VEGF therapies, panretinal photocoagulation, steroids, and tetracycline derivatives are not yet well investigated but clearly may play a role in how patients respond to these treatments. Studies have focused on microglia behavior under light-induced retinal damage and laser treatments and the role that anti-VEGF treatments have on retinal microglia. Although microglia are activated and migrate toward the site of insult, the effects of the morphological and phenotypic changes of activated microglia on the damaged and surrounding tissue remain unknown. Additionally, to appreciate the effects of these treatments on retinal microglia under disease conditions, the presence of activated microglia due to the initial insult and prior to treatments should be considered in future studies. Available studies performed to determine the effect of anti-VEGF treatments have shown a decrease in activated microglia due to treatment. However, these studies have been performed in the absence of diabetic insult. Retinal photocoagulation has been demonstrated to induce activation of microglia, but variable phenotypes have been observed. The roles of the different phenotypes of activated microglia observed after retinal photocoagulation have yet to be determined. Additionally, it is good to remember that the functions of microglia do not happen in isolation but likely influence and are influenced by neighboring cells such as astrocytes, neurons, and the blood-retina barrier. Investigators must determine how DR treatments affect the actions of microglia and in turn how the actions of microglia can be modified to enhance the benefits of treatments.

## Figures and Tables

**Table 1:** Distribution of microglia and macrophages in retinal layers.

Location	NFL	GCL	IPL	INL	OPL	ONL	PR	SS	RPE	CHD	SCL
Vascular network	X	X	X	X						X	X
Early MG	X	X									
Adult MG	X	X	X	X							
Inflammation											
MG	X	X	X	X	X	X	X	X			
Macrophages	X	X						X	X	X	

Key: MG: microglia; OPL: outer plexiform layer; ILM: inner limiting membrane; ONL: outer nuclear layer; NFL: nerve fiber layer; PR: photoreceptor layer; GCL: ganglion cell layer; SS: subretinal space; IPL: inner plexiform layer; RPE: retinal pigment epithelium; INL: inner nuclear layer; CHD: choroid; and SCL: sclera.

Based on findings from human, primate, rats, mice, and quail retinas. Adapted from Boycott and Hopkins, 1981, cited by Chen et al., 2002 [10], Santos et al., 2008 [11], Cuadros and Navascués, 1998 [12], and Tan et al., 2012 [13]

**Table 2:** Distribution of microglia in healthy eyes [64].

	Retinal parenchyma	Inner retinal layers	Perivascular space
CD-45	X	X	
CD-68			X
HLA-DR	X	X	

**Table 3:** Distribution of microglia in background diabetic retinopathy [64].

	ILM	RNFL	GCL	IPL	INL	OPL	ONL	OLM	PR	RPE	Endo cell	ERM	Fresh heme/MA	Arterioles venules	Capillaries	Retinal parenchyma
CD-45	X	X	X	X	X							X		X	X	
CD-68	X	X	X	X	X							X		X	X	
HLA-DR	X	X	X	X	X	X	X		X		X	X	X	X	X	X

ERM: Epi-rental membrane.

**Table 4:** Distribution of microglia in preproliferative diabetic retinopathy [64].

	ILM	RNFL	GCL	IPL	INL	OPL	ONL	OLM	PR	RPE	Cotton wool spots	Dilated venules	Pial septa ONH
CD-45	X	X	X	X	X						X	X	X
CD-68	X	X	X	X	X						X	X	X
HLA-DR	X	X	X	X	X						X	X	X

ONH: Optic nerve head.

**Table 5:** Distribution of microglia in proliferative diabetic retinopathy [64].

	ILM	RNFL	GCL	IPL	INL	OPL	ONL	OLM	PR	RPE	Neovascularization area of the vitreous	Central Kuhnt meniscus ONH
CD-45		X									X	X
CD-68		X									X	X
HLA-DR		X									X	X

ONH: Optic nerve head.

**Table 6:** Retinal Cell Cytokines.

Cytokine	Condition∗	References
IL-1*β*	*In vitro*, primary microglia culture; LPS and minocycline treated; culture medium quantified (rat)	Wang et al. 2005 [57]
IL-1*β*	*Ex vivo*, STZ treated; LPS and minocycline treated; whole retina homogenate (rat)	Liu et al. 2012 [90, 91]; Yang et al. 2009 [42]; Krady et al. 2005 [56]
IL-3	*In vivo*, vitreous quantified (mouse)	Liu et al. 2012 [90, 91]
IL-6	*In vivo*, vitreous quantified (mouse and human)	Liu et al. 2012 [90, 91]; Abcouwer 2013 [69]
IL-8	*In vivo*, vitreous quantified (mouse and human)	Liu et al. 2012 [90, 91]; Abcouwer 2013 [69]
IL-10	*In vivo*, vitreous quantified (mouse)	Liu et al. 2012 [90, 91]
IL-12	*In vivo*, vitreous quantified (mouse)	Liu et al. 2012 [90, 91]
IL-18	*Ex vivo*, STZ treated; whole retina homogenate (rat)	Yang et al. 2009 [42]
TNF-*α*	*In vitro*, primary microglia culture; LPS and minocycline treated; AGE treated; culture medium quantified (rat)	Wang et al. 2005 [57]; Krady et al. 2005 [56]
TNF-*α*	*Ex vivo*, LPS and minocycline treated; STZ treated; whole retina homogenate (rat)	Yang et al. 2009 [42]; Krady et al. 2005 [56]

*The condition column represents how cytokine level was quantified, i.e., culture medium collected; whole retina homogenate; or vitreous humor collected and assayed for cytokine production.
